# Expression and potential regulatory functions of *Drosophila* octopamine receptors in the female reproductive tract

**DOI:** 10.1093/g3journal/jkae012

**Published:** 2024-01-19

**Authors:** Ethan W Rohrbach, Elizabeth M Knapp, Sonali A Deshpande, David E Krantz

**Affiliations:** Interdepartmental Program in Neuroscience, Brain Research Institute, Gonda (Goldschmied) Neuroscience and Genetics Research Center, UCLA, Los Angeles, CA 90095, USA; Department of Psychiatry and Biobehavioral Sciences, David Geffen School of Medicine, UCLA, Los Angeles, CA 90095, USA; Department of Psychiatry and Biobehavioral Sciences, David Geffen School of Medicine, UCLA, Los Angeles, CA 90095, USA; Department of Psychiatry and Biobehavioral Sciences, David Geffen School of Medicine, UCLA, Los Angeles, CA 90095, USA

**Keywords:** octopamine, octopamine receptor, egg-laying, spermatheca, oviposition

## Abstract

Aminergic signaling is known to play a critical role in regulating female reproductive processes in both mammals and insects. In *Drosophila*, the ortholog of noradrenaline, octopamine, is required for ovulation as well as several other female reproductive processes. Two octopamine receptors have already been shown to be expressed in the *Drosophila* reproductive tract and to be required for egg-laying: *OAMB* and *Octβ2R*. The *Drosophila* genome contains 4 additional octopamine receptors—*Octα2R*, *Octβ1R*, *Octβ3R*, and *Oct-TyrR*—but their cellular patterns of expression in the reproductive tract and potential contribution(s) to egg-laying are not known. In addition, the mechanisms by which *OAMB* and *Octβ2R* regulate reproduction are incompletely understood. Using a panel of MiMIC Gal4 lines, we show that *Octα2R*, *Octβ1R*, *Octβ3R*, and *Oct-TyrR* receptors are not detectable in either epithelium or muscle but are clearly expressed in neurons within the female fly reproductive tract. Optogenetic activation of neurons that express at least 3 types of octopamine receptors stimulates contractions in the lateral oviduct. We also find that octopamine stimulates calcium transients in the sperm storage organs and that its effects in spermathecal, secretory cells, can be blocked by knock-down of *OAMB*. These data extend our understanding of the pathways by which octopamine regulates egg-laying in *Drosophila* and raise the possibility that multiple octopamine receptor subtypes could play a role in this process.

## Introduction

The regulation of some reproductive processes is surprisingly conserved, allowing the use of relatively simple systems to explore the underlying mechanisms: these include the aminergic regulation of oocyte development and ovulation mediated by noradrenalin in mammals and its structural ortholog octopamine (OA) in *Drosophila melanogaster* (White et al. 2021b) (Kim et al. 2021) (Deady and Sun 2015) (Andreatta et al. 2018) (Meiselman et al. 2018) *(*Yoshinari et al. 2020*). D. melanogaster* expresses 6 OA receptors (El-Kholy et al. 2015) (Qi et al. 2017) (Evans and Maqueira 2005) (Balfanz et al. 2005) (Maqueira et al. 2005), and 2 of them, *OAMB* and *Octβ2R*, have been proposed to regulate egg-laying and female fertility (Lee et al. 2003) (Lee et al. 2009) (Lim et al. 2014) (Li et al. 2015). *OAMB* is expressed in a sheath of follicle cells that surround the egg and is required for OA-dependent rupture of the sheath (Deady and Sun 2015). Both *Octβ2R* and *OAMB* are expressed in epithelial cells that line the oviducts, and expression of *OAMB* at this site is required for fertility (Lee et al. 2003) (Lee et al. 2009) (Lim et al. 2014) (Deshpande et al. 2022). *OAMB* also regulates the function of the seminal receptacle and spermathecae (Avila et al. 2012). Both *OAMB* and *Octβ2R* are expressed in central and peripheral neurons that innervate the reproductive tract, and *Octβ2R*-expressing neurons in the reproductive tract may regulate oviduct contractions (Deshpande et al. 2022). Surprisingly, despite the proposed roles of both *OAMB* and *Octβ2R* in muscle contractility, neither is detectably expressed in muscle using the MiMIC GAL4 lines that we have used (Deshpande et al. 2022). In addition, while some of the signaling pathways responsible for the effects of *OAMB* and *Octβ2R* are known, others, including the mechanism by which *OAMB* regulates sperm storage, remain incompletely understood.

*Drosophila* express 4 other OA receptors in addition to *OAMB* and *Octβ2R*: *Octα2R*, *Octβ1R*, *Octβ3R*, and *Oct-TyrR* (El-Kholy et al. 2015) (Qi et al. 2017) (Evans and Maqueira 2005) (Balfanz et al. 2005) (Maqueira et al. 2005). At the cellular level, their patterns of expression in the reproductive tract are not known (El-Kholy et al. 2015) and it is not known whether they may play any role in reproduction. We show here that *Octα2R*, *Octβ1R*, *Octβ3R*, and *Oct-TyrR* are expressed in neurons and neuronal processes in the reproductive tract, but unlike *OAMB* and *Octβ2R*, they are not expressed in epithelial cells. To further probe the potential function of OA in the reproductive tract, we have tested the effects of optogenetically stimulating OA receptor–expressing cells and the effects of OA on the acute regulation of calcium transients in the sperm storage organs.

## Materials and methods

### Experimental model and subject details

*D. melanogaster* were raised in mixed sex vials on cornmeal/sucrose/yeast/sucrose/dextrose/agar medium at 25°C and 50–70% humidity under a 12:12 light:dark cycle unless otherwise noted. All experiments used mated or virgin female flies 4–6 days post eclosion. All fly lines used in this study are listed in Table 1.

**Table 1.** Fly lines and antibodies used in this study.

Reagent type	Symbol	Identifier	Source	Full genotype
*Drosophila* line	OAMB-T2A-Gal4	#84675	BDSC	y[1], w[*]; Mi{Trojan-GAL4.1}Oamb[MI12417-TG4.1]
	Octα2R-T2A-Gal4	#84610	BDSC	y[1] w[*]; Mi{Trojan-GAL4.0}Octalpha2R[MI10227-TG4.0]/TM3, Sb[1] Ser[1]
	Octβ1R-T2A-Gal4	#86139	BDSC	TI{RFP[3xP3.cUa]=2A-GAL4}Octbeta1R[2A-C.GAL4.KI]/TM3, Sb[1]
	Octβ2R-T2A-Gal4	#84678	BDSC	w[*]; TI{2A-GAL4}Octbeta2R[2A-ACDE.GAL4]
	Octβ3R-T2A-Gal4	#84680	BDSC	w[*]; TI{2A-GAL4}Octbeta3R[2A-FG.GAL4]/TM3, Sb[1]
	Oct-TyrR-T2A-GAL4	#86138	BDSC	TI{RFP[3xP3.cUa]=2A-GAL4}Oct-TyrR[2A-GAL4.KI]
	UAS-mCD8::GFP	#32194	BDSC	w[*]; P{y[+t7.7] w[+mC]=20XUAS-IVS-mCD8::GFP}attP2
	Ppk1.0-LexA	N/A	Bing Ye	w ; Sp / CyO ; Ppk1.0-LexA / TM6
	UAS-mCD8::GFP, LexAop-CD2::RFP	#67093	BDSC	y[1] w[*]; P{y[+t7.7] w[+mC]=lexAop-rCD2::RFP-p10.UAS-mCD8::GFP-p10}su(Hw)attP5/CyO; TM3, Sb[1]/TM6B, Tb[1]
	UAS-ChR2-XXM	N/A	Robert Kittel	w[1118]; UAS-CHR2(XXM)::tdTomato / CyO
	24B-Gal4	#1767	BDSC	w[*]; P{w[+mW.hs]=GawB}how[24B]
	40B09-Gal4	#41235	BDSC Jianjun Sun	w[1118]; P{y[+t7.7] w[+mC]=GMR40B09-GAL4}attP2
	UAS-RCaMP1b	#63793	BDSC	w[*]; PBac{y[+mDint2] w[+mC]=20XUAS-IVS-NES-jRCaMP1b-p10}VK00005
	UAS-OAMB-RNAi	#31233	BDSC	y[1] v[1]; P{y[+t7.7] v[+t1.8]=TRiP.JF01732}attP2
	UAS-dicer2	#24651	BDSC	w[1118]; P{w[+mC]=UAS-Dcr-2.D}10
Antibodies/labeling reagents	Mouse anti-GFP	Cat# A11120	Invitrogen	
	AF488-conjugated goat-anti-mouse	Cat# A28175	Invitrogen	
	AF555-conjugated phalloidin	Cat# A34055	Invitrogen	
	Rabbit anti-dsRed	Cat# 632496	Takara Bio	
	AF568-conjugated goat-anti-rabbit	Cat# A11036	Invitrogen	

### Fly husbandry and stocks

Publicly available fly lines with noted identifiers were identified using Flybase (Gramates et al. 2022) obtained from the Bloomingtom *Drosophila* Stock Center (BDSC) (listed in Table 1). We would like to thank Jianjun Sun (University of Connecticut) for information on the spermatheca expression of the *40B09-Gal4* allele. We also thank the following people for generously supplying the following additional lines: Bing Ye (University of Michigan) for *ppk1.0-LexA* (Gou et al. 2014), Soo Hong Min (Harvard), and Robert Kittel (University of Würzberg) for *UAS-ChR2-XXM* (Dawydow et al. 2014) (Scholz et al. 2017).

### Immunofluorescent labeling

To visualize the expression patterns of the OA receptor genes, the reproductive systems from flies harboring *UAS-mCD8-GFP* and OA receptor *MiMIC-T2A-Gal4*s were dissected in phosphate buffered saline (PBS) at 25°C with the ventral nerve cord (VNC) and medial abdominal nerve (MAN) connection left intact. The tissue was fixed in 4% paraformaldehyde (PFA) for 15 min and washed in PBS containing 0.3% (vol/vol) Triton X-100 (PBT) for 30 min. Following fixation, preparations were washed for 1 h in blocking buffer containing 5% (vol/vol) normal goat serum (NGS) (Cat# G9023, Sigma-Aldrich) in PBT then incubated for 24–48 h at 4°C in the primary antibody mouse anti-GFP (1:500 in blocking buffer, Cat# A11120, Invitrogen). The sample was washed in PBT for 3 h at 25°C then incubated in the secondary antibody AF488-conjugated goat-anti-mouse (1:500 in blocking buffer, Cat# A28175, Invitrogen) at 4°C for 24 h. After washing in PBT for 1 h at 25°C, the preparations were optically cleared in 25% glycerol for 1 h at 25°C and then mounted on Superfrost slides (Cat# 12-550-143, Fisherbrand) with bridged glass cover slips #0 (Cat# 72198-10, Electron Microscopy Sciences) in Fluoromount-G mounting media (Cat# 0100-01, SouthernBiotech). For colabeling of muscle cells, preparations were dissected and processed as described above, except AF555-conjugated phalloidin (1:500, Ref# A34055, Invitrogen) was included in the secondary antibody solution. For colabeling of *ppk1.0-LexA*, preparations were dissected and processed as described above, except rabbit anti-dsRed (1:500, Cat# 632496, Takara Bio) was added to the primary antibody solution and AF568-conjugated goat-anti-rabbit (1:500, Cat# A11036, Invitrogen) was added to the secondary antibody solution.

### Optogenetic stimulation of lateral oviduct contractions

For optogenetic experiments, mated female flies were raised in standard food containing 80 μM all-trans retinal from 1 day post eclosion until tested (5–7 days). To minimally disrupt nerves in and around the reproductive tract, optogenetic stimulations of OA receptor-expressing cells were performed on “abdominal fillet” preparations as previously described (Deshpande et al. 2022). In brief, the abdomen was separated from the rest of the fly body using microscissors and pinned to a Sylgard dish, and the sternal plates removed to expose the reproductive organs. Stimulation was performed using a Lambda DG-4 light source (Sutter), Chroma filter set 41001, and the light path of an Axio Examiner Z1 microscope to illuminate the entire field of view at 1 mW/mm^2^ power. The preparation was also illuminated from the side using an external LED to facilitate the visualization of contractions. Contractions of the LO were defined by a decrease in the distance between ovaries and a characteristic contraction of the oviduct tissue as described (Deshpande et al. 2022) and counted in video recordings acquired with a charge-coupled device (CCD) camera (Andor iXon 897, Oxford Instruments, Oxfordshire, England) at a capture rate of 12 frames/s and using Andor IQ2 software. Flies harboring 1 copy of *Octβ1R*, *Octβ3R*, or *Oct-TyrR wMiMIC-T2A-Gal4* and 1 copy of *UAS-ChR2-XXM::tdTomato* were compared with controls that expressed *UAS-ChR2-XXM::tdTomato* alone.

### Live imaging of sperm storage organs

To specifically determine OA's effects on the accessory gland cellular activity, without simultaneously activating peripheral circuits associated with the reproductive tract, we performed live imaging using a previously described “isolated preparation” (Deshpande et al. 2022). The reproductive tract was dissected out of the abdomen, and the abdominal cuticle, the gut, and fat bodies were removed. The MAN was cut and the CNS was also removed. The anterior tip of the ovaries and the distal end of the uterus were pinned to a Sylgard substrate with insect pins. Live imaging of RCaMP1b expressed in the seminal receptacle and spermathecae was performed using a 555 nm LED light source (Thorlabs), the standard Chroma filter set 41007a, and a Zeiss Achroplan water immersion 10× objective on a Zeiss Axio Examiner Z1 microscope with a CCD camera (Andor iXon 897, Oxford Instruments, Oxfordshire, England) at a capture rate of 12 frames/s using Andor IQ2 software. Images were analyzed using Fiji/ImageJ software (Schindelin et al. 2012). For all regions of interest (ROIs), an off-target area of equal size was selected as background. In all experiments, 1 min of baseline activity was recorded before OA was bath applied to the preparation at the indicated concentration. Changes in fluorescence are reported as the background-subtracted difference in the change in fluorescence divided by baseline {ΔF/F = [(F peak − F baseline)/F baseline], where F baseline = average RCaMP signal during the 1 min before OA bath application}. Recording was conducted for 4 min following OA addition. All flies tested were 4 days post eclosion and carried either 1 copy of *24b-Gal4* or *40B09-Gal4* and 1 copy of *UAS-RCaMP1b*. RNAi experiments used flies that additionally included 1 copy each of *UAS-dicer2* and *UAS-OAMB-RNAi*. Mated flies were cohoused with *CS* males after sorting 1 day post eclosion. Virgin flies were sorted and housed without males.

### Statistical analysis

The optogenetic data shown in Fig. 8 were analyzed using Dunnett's test in the base R package. The data in Fig. 8g–h were analyzed using 2-way ANOVA in GraphPad Prism.

## Results

### All OA receptors are expressed in the reproductive tract

**Fig. 1.**
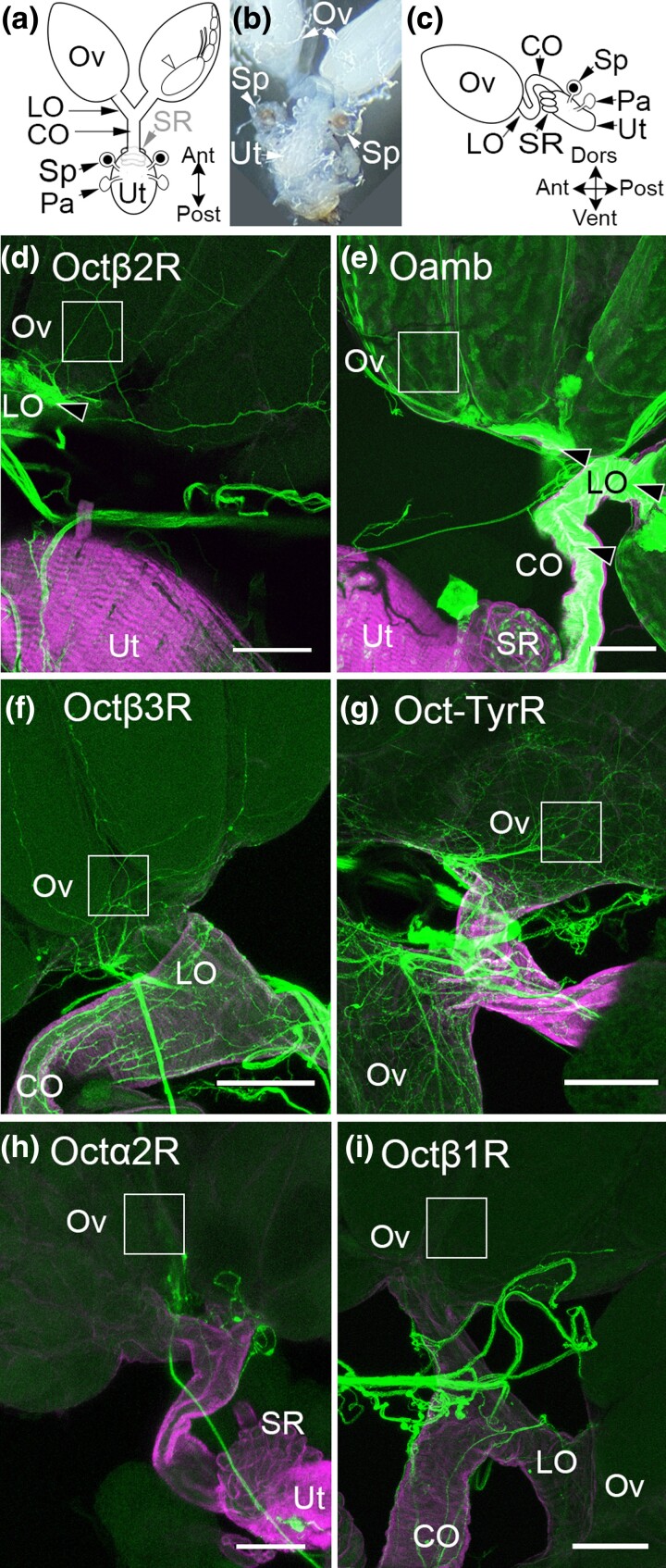
OA receptor expression in the ovaries. a) A schematized dorsal view of the female Drosophila reproductive tract showing a mature egg (white arrowhead), the ovaries (Ov), lateral oviducts (LO), common oviduct (CO), and the uterus (Ut). Two sperm storage organs are attached to the uterus: the seminal receptacle (SR, indicated in gray because it is on the ventral side of the Ut) and the 2 spermathecae (Sp). An additional pair of parovarian/accessory glands (Pa) is attached to the uterus posterior to the spermatheca. b) A dissected preparation viewed from the dorsal side showing the relative sizes and positions of the Ov, Sp, and Ut. c) A schematize sagittal view of the reproductive tract as it appears in vivo. d–i) MiMIC Gal4 lines for the indicated receptors were used to express membrane-attached GFP (green) and colabeled for phalloidin (magenta) as a marker for muscle cells. The boxed areas highlight the presence or absence of fine processes that ascend into the ovaries. Faint labeling of follicle cells within the boxed area can be seen with OAMB (e). Labeling of the oviduct epithelium is also visible for Octβ2 and OAMB (black arrowheads). Scale bars: 100 μm.

Using a panel of *MiMIC-T2A-Gal4* lines, each 1 of the 6 *Drosophila* OA receptors has previously been shown to be expressed in the fly central nervous system (McKinney et al. 2020). We previously used these lines to investigate the expression and function of *Octβ2R* and *OAMB in* the reproductive tract, focusing on the oviducts (Deshpande et al. 2022). We have now extended the analysis of *Octβ2R* and *OAMB* to other regions of the reproductive tract and used additional MiMIC lines to determine the expression patterns of *Octα2R*, *Octβ1R*, *Octβ3R*, and *Oct-TyrR* (Fig. 1a–i). We observe fine processes at the base of the ovaries that express *Octβ2R* (Fig. 1d) but not *OAMB* (Fig. 1e). *Octβ3R-* and *Oct/TyrR*-expressing processes also extend into the ovary (Fig. 1f and g), but we do not detect expression of Oct*βR-* or *Octα2R*-expressing processes in this region (Fig. 1h and i).

For *Octβ2*, some labeled processes in the reproductive tract represent autoreceptors expressed in Tdc2(+) neurons as seen in colabeling experiments for *Tdc2* and *Octβ2R* (Deshpande et al. 2022). In contrast to *Octβ2R*, we do not detect coexpression of *Octβ3R* or *Oct-Tyr*R in *Tdc2*(+) neurons (data not shown). Therefore, the *Octβ3R* and *Oct-TyrR* processes that we detect in the ovaries and elsewhere in the reproductive tract represent other types of neurons that remain to be identified.

Consistent with previous reports (Lee et al. 2003) (Deady and Sun 2015), we detect *OAMB* in follicle cells that surround the mature oocyte (Fig. 1e, boxed area). In addition, we detect a swath of labeling at the base of the ovaries; this may represent the insect analog of the corpus luteum that consists of follicle cells that have been shed from the activated oocyte (data not shown) (Deady and Sun 2015). We do not detect expression of any other OA receptors in the follicle cells (Fig. 1 and data not shown).

**Fig. 2.**
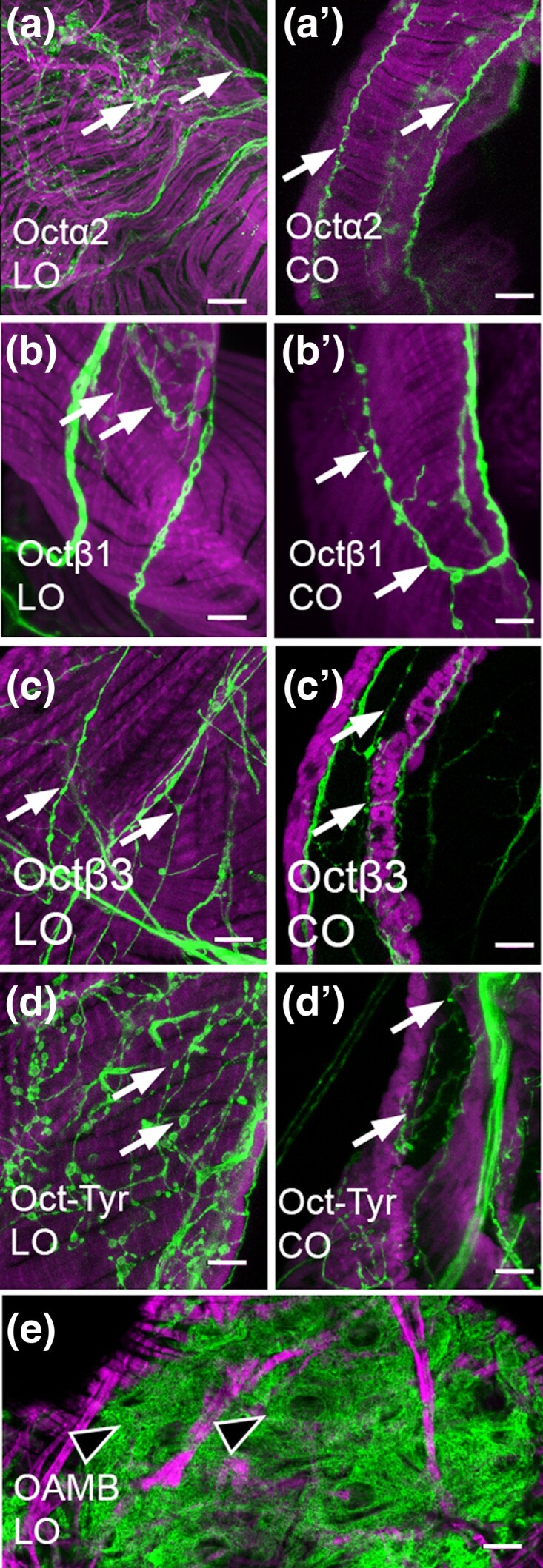
Processes in the oviducts. a–e). MiMIC Gal4 lines for the indicated receptors were used to express membrane-attached GFP (green) and colabeled for phalloidin (magenta) as in Fig. 1. Fine processes (white arrows) are present for *Octα2R*, *Octβ1R*, *Octβ3R*, and *Oct-TyrR* as previously reported for *Octβ2R* and OAMB. Labeling in muscle or epithelium was not detected. An example of epithelial cells expressing OAMB is shown (e, black arrowheads). Scale bars: 10 μm.

**Fig. 3.**
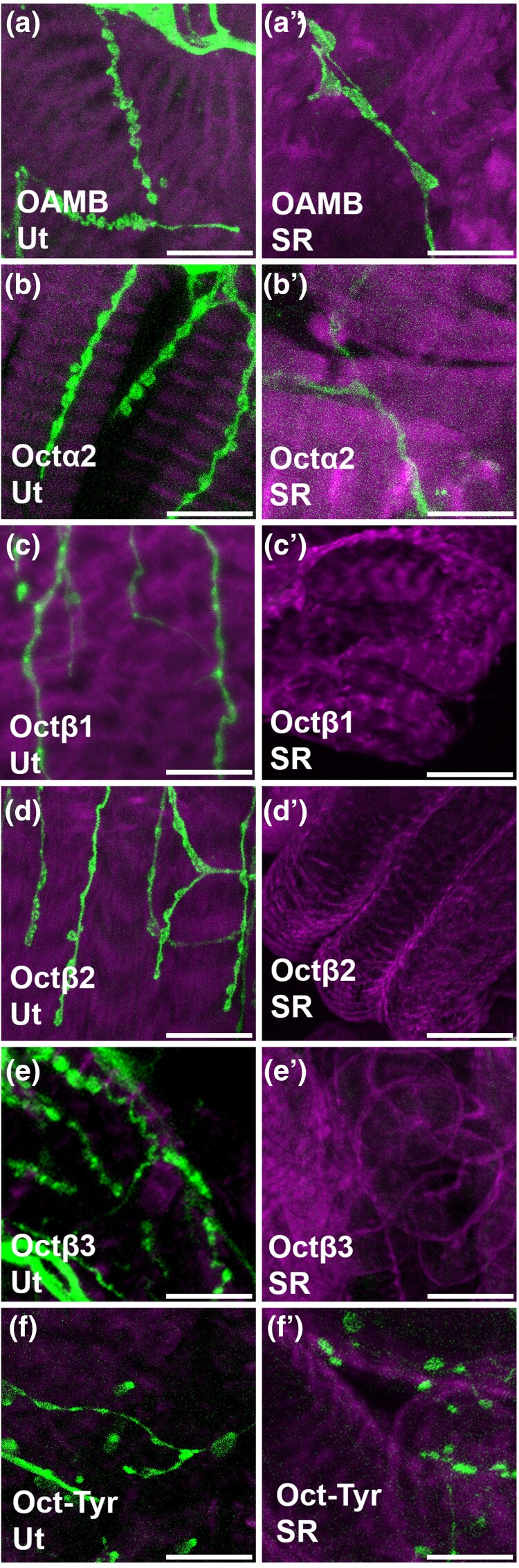
Processes in the uterus and seminal receptacle. a–f) MiMIC Gal4 lines for the indicated receptors were used to express membrane-attached GFP (green) and colabeled for phalloidin (magenta) as in Figs. 1 and 2. Processes in the uterus (a–f) are visible for all receptors. Processes in the seminal receptable (a′–f′) are visible for *OAMB*, *Octα2R*, and *Oct-TyrR* but not *Octβ1R*, *Octβ2R*, or *Octβ3R*. Scale bars: 10 μm.

Both *OAMB* and *Octβ2* are expressed in the epithelial cells that line the lumen of the oviducts (Lee et al. 2003) (Deshpande et al. 2022), and genetic rescue studies have indicated that both may function in the epithelium to promote egg-laying (Lim et al. 2014). In contrast to *OAMB* and *Octβ2*, we do not detect expression of *Octα2R*, *Octβ1R*, *Octβ3R*, or *Oct-TyrR* in the epithelial cells that line the oviducts (Fig. 2). For comparison, an example of epithelial cells in the LO expressing OAMB is shown in Fig. 2e. Similar to both *Octβ2R* and *OAMB*, all other OA receptors including *Octα2R*, *Octβ1R*, *Octβ3R*, and *Oct-TyrR* can be detected in fine processes within both the LO and CO (Fig. 2a–d). Processes that express all 6 OA receptors are also present in the uterus, while the SR is innervated only by *OAMB*-, *Octα2R*-, and *Oct/TyrR*-expressing cells (Fig. 3). A summary of these and all other expression data are shown in Table 2.

**Table 2.** Data summary.

		OAMB	Octα2R	Octβ1R	Octβ2R	Octβ3R	Oct-TyrR
CNS neurons		+	+	+	+	+	+
Ovarian follicle cells		+	−	−	−	−	−
Epithelium	LO	+	−	−	+	−	−
	CO	+	−	−	−	−	−
Presumptive secretory cells	Spermatheca	+	−	−	−	−	−
	Parovarian gland	+	−	−	−	−	−
Innervation	Ovaries	−	−	−	+	+	+
	LO	+	+	+	+	+	+
	CO	+	+	+	+	+	+
	Ut	+	+	+	+	+	+
	SR	+	+	−	−	−	+
Peripheral neurons	Anterior Ut	+	+	−	+	+	−
	Posterior Ut	+	−	+	−	−	−
	“Free-hanging”	−	+	+	−	+	+
ChR2-induced oviduct contractions		−	ND	+	+	+/−	+

The expression of the receptor GAL4 MiMIC lines at each site and the response of the oviducts to optogenetic (ChR2-induced) stimulation are indicated.

ND, not determined.

### Peripheral cell bodies express OA receptors

**Fig. 4.**
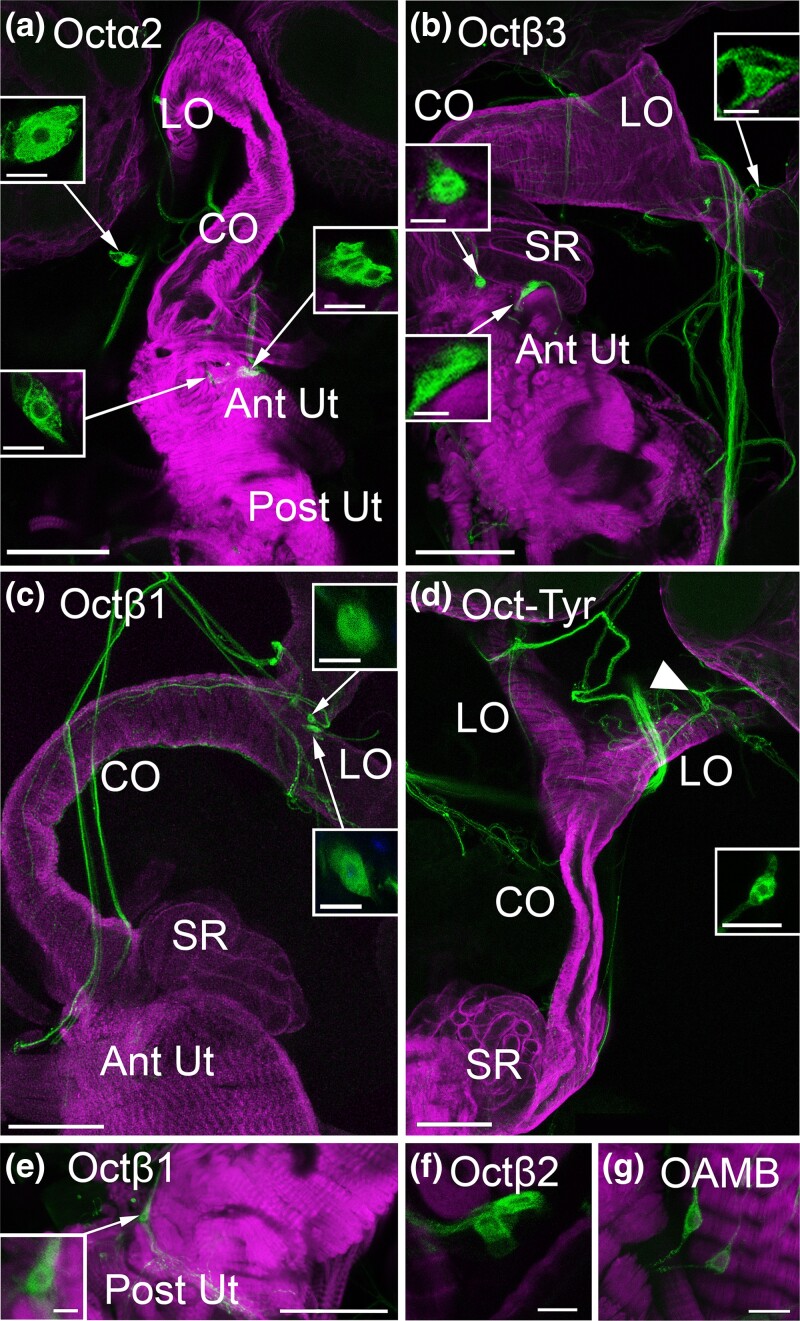
Peripheral cell bodies in the reproductive tract. a–g) MiMIC Gal4 lines for the indicated receptors were used to express GFP (green) and colabeled for phalloidin (magenta) as in Figs. 1–3. Cell bodies in the anterior uterus (Ant Ut) express *Octα2R* (a) and *Octβ3R* (b), and at least 1 cell body expressing *Octβ1R* is present in the posterior uterus (e). All other cell bodies shown here that express *Octα2R*, *Octβ3R*, *Octβ1R*, and *Oct-TyrR* are contained within or attached to free-hanging nerves that connect the MAN to the reproductive tract or connect the anterior and posterior regions of the reproductive tract (a–d). Previously reported cells expressing *Octβ2R* in the anterior uterus (f) and *OAMB* in the posterior uterus are shown for comparison (g). Boxed insets show the indicated cells at a higher magnification. Scale bars: (a–e) 100 μm; (f and g and boxed insets in a–d) 10 μm.

Some processes from *Octβ2R* and *OAMB* are likely to be derived from neurons in the VNC (McKinney et al. 2020), and labeled processes for all 6 OA receptors can be detected in the medal abdominal nerve (MAN) that connects the VNC to the reproductive tract (data not shown). We have previously reported that a small number of peripheral neurons intrinsic to the reproductive tract also express *Octβ2R* and *OAMB*, some of which localize to the uterus (Deshpande et al. 2022). Similarly, we detect a small cluster of cell bodies in the anterior uterus that expresses *Octα2R* and *Octβ3R* (Fig. 4a and b). We do not detect *Octβ1R(+)* cells in the anterior uterus (Fig. 1c), but at least 1 *Octβ1R(+)* cell localizes to the posterior uterus (Fig. 4e). For comparison, we show subsets of cells that express *Octβ2R* in the anterior uterus (Fig. 4f) and *OAMB* in the posterior uterus (Fig. 4g) as previously reported (Deshpande et al. 2022). OA receptor–expressing cell bodies are also present within the nerves that descend from the AbG and that connect the anterior uterus to the base of the ovaries (indicated as free-hanging in Table 2 and Fig. 4). These include cells present within bilaterally symmetric nerves near the oviducts that express *Octα2R*, *Oct-TyrR*, *Octβ1R*, and *Octβ3R* (Fig. 4a–d).

**Fig. 5.**
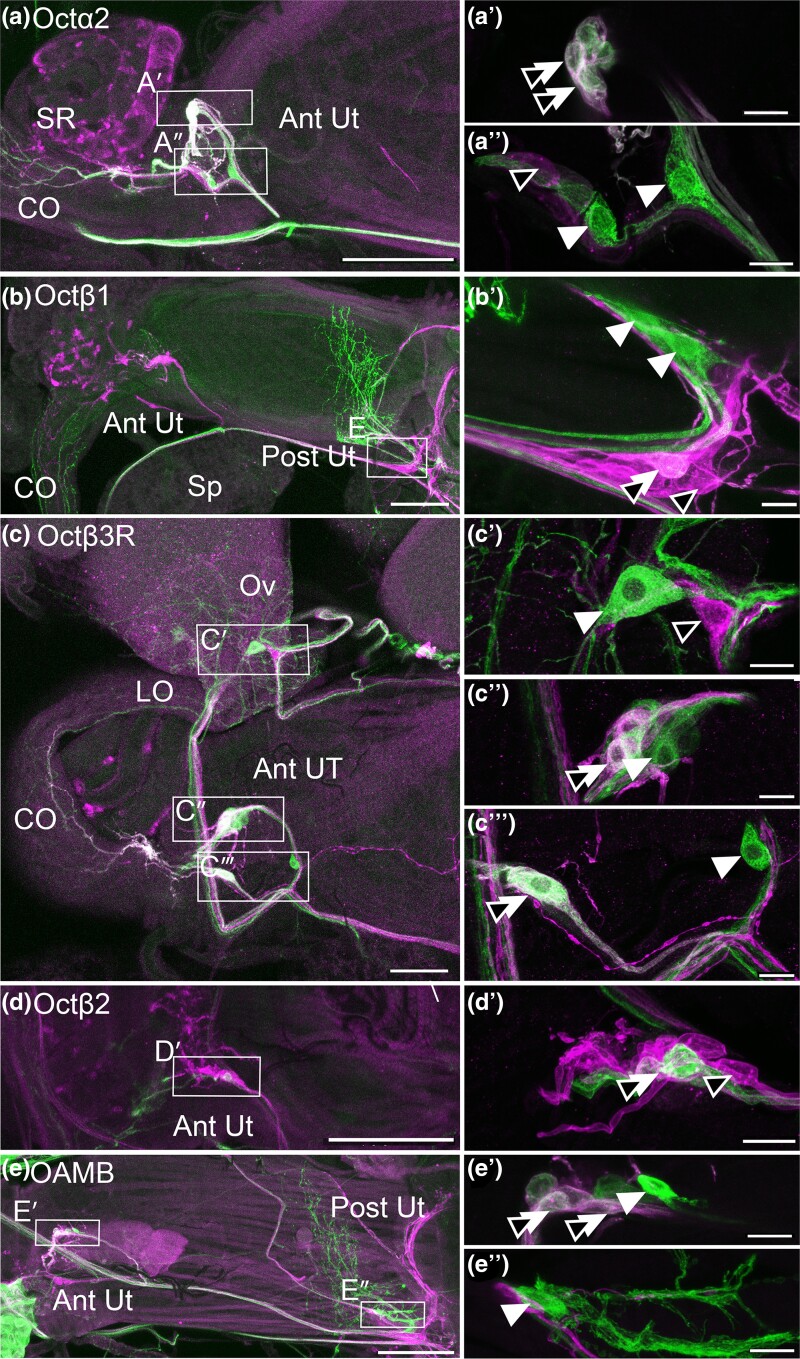
A subset of peripheral cell bodies expressing OA receptors colabel with the marker *ppk1.0-LexA*. a–e) MiMIC Gal4 lines for the indicated receptors were used to express GFP (green) and colabeled with *ppk1.0-LexA* expressing RFP (magenta). The boxed regions indicated in a–e are shown at a higher magnification in a′–e′ and a″−e″. Cells labeled for the receptor alone (white arrowhead), *ppk1.0* alone (black arrowhead), or both the receptor and *ppk1.0* (double white + black arrowheads) are indicated. Scale bars: (a–e) 100 μm; (a′–e′, a″−e″) 10 μm.

We have previously shown that both *OAMB* and *Octβ2R* are coexpressed in neurons that express *ppk1.0-LexA* (Deshpande et al. 2022), a marker for mechanosensitive neurons that regulate CO contractions and postmating behavior (Gou et al. 2014) (Hasemeyer et al. 2009). We find that subsets of cells in the anterior uterus that express *Octα2R* or *Octβ3R* coexpress ppk1.0-LexA (Fig. 5a and c). In addition, at least 1 *Octβ1*(+) cell in the posterior uterus expresses ppk1.0-LexA (Fig. 5b). The coexpression of *Octβ2R* and *OAMB* with *ppk1.0-LexA* is shown for comparison (Fig. 5d and e).

### OAMB is expressed in the sperm storage organs and accessory glands

**Fig. 6.**
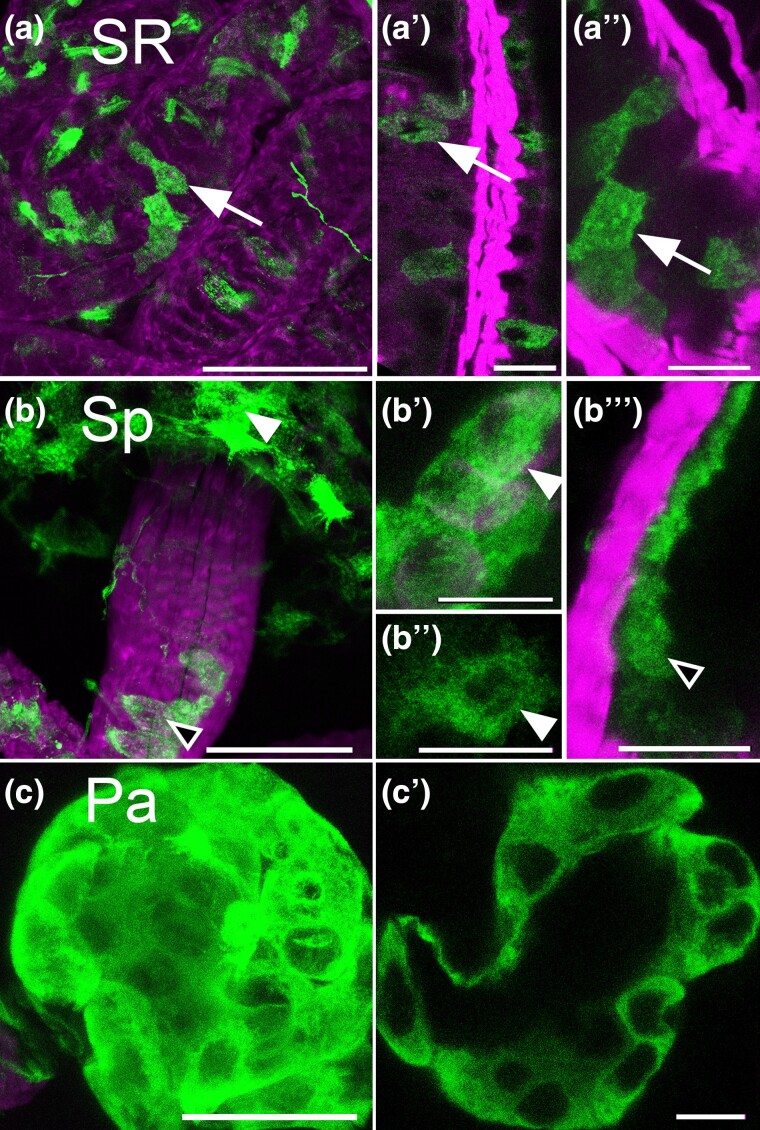
*OAMB* is expressed in the seminal receptacle and spermathecae. a–c) *OAMB(+) cells* were marked with *GFP* and colabeled with phalloidin (magenta). Cells in the seminal receptacle (a–a″, SR, white arrows), the spermathecae (b–b′′′, Sp), and the parovarion glands (c–c″, Pa) express *OAMB*. In the spermathecae (b–b′′′), cells were labeled in both the bulb (white arrowheads) and the lumen of the stalk (black arrowheads). Scale bars: (a–c) 50 μm; (a–a″, b–b′′′, and c′) 10 μm.

OA has been shown to regulate sperm storage, and RNA knock-down experiments have implicated OAMB in this process (Avila et al. 2012). We observe expression of the *OAMB-Gal4* MiMIC in the seminal receptacle, the spermatheca, and the parovarian glands, consistent with a previous report using a custom Ab and additional tissue-specific Gal4 lines (Lee et al. 2003) (Fig. 6). We do not detect any additional OA receptors in the bulb of the spermathecae or the “cap” of the parovarian glands (data not shown). The location of the *OAMB*(+) cells in the bulb of the spermathecae is consistent with that of previously described secretory cells (Schnakenberg et al. 2011) (Allen and Spradling 2008). The identity of the cells that express *OAMB* in the lumen of the spermathecae and seminal receptacle is not clear; however, their location and shape suggest the possibility that they are a component of an epithelial like-layer, perhaps similar to that which lines the oviducts.

### Optogenetic stimulation of OA receptor-expressing cells drives lateral oviduct muscle contractions

**Fig. 7.**
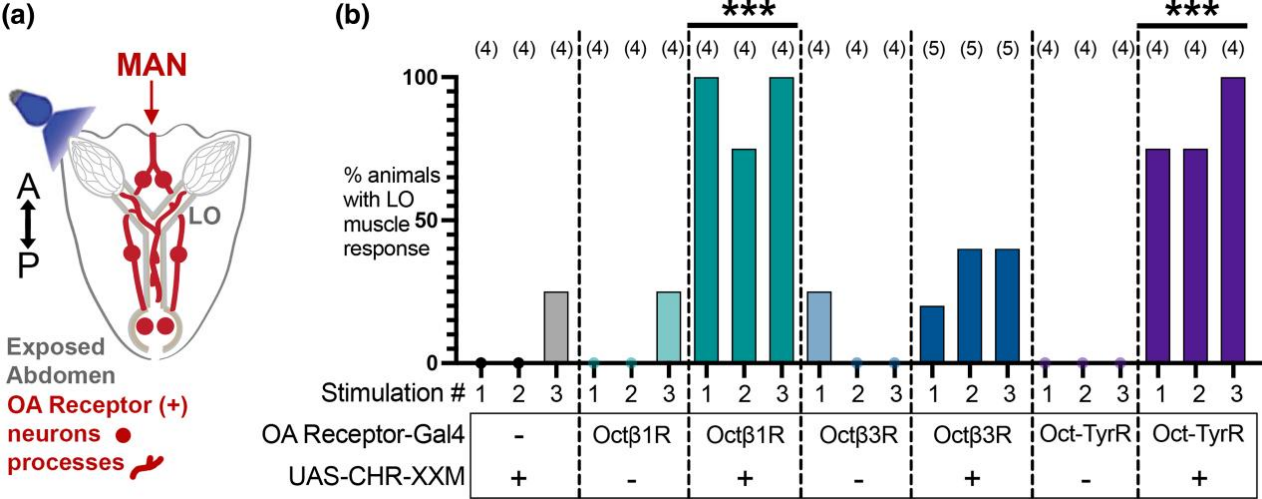
Optogenetic stimulation of OA receptor–expressing cells drives lateral oviduct contractions. a) Fly abdomens were severed from the throrax, dissected to expose the reproductive organs, and optogenetically stimulated (b). The number of LO contractions in response to each of 3 successive stimulations per fly was quantified. The number of flies per genotype is indicated in parentheses (*n*). The data were analyzed using Dunnett's test to compare multiple conditions to the negative control *UAS-ChRXXM* without a Gal4 driver. The control differed significantly (***P < 0.001) from flies expressing *UAS-ChRXXM* with *Octβ1R-Gal4* or *Oct-TyrR-Gal4* but not *Octβ3R-Gal4* or flies expressing a Gal4 driver in the absence of *UAS-ChRXXM*.

We have previously shown that optogenetic stimulation of cells that express *Octβ2R* induces lateral oviduct contractions (Deshpande et al. 2022). To determine whether other OA receptor–expressing cells might also promote oviduct contractility, we expressed the channelrhodopsin variant *ChR2-XXM* (Dawydow et al. 2014) (Scholz et al. 2017) using other OA receptor MiMIC Gal4 lines. In addition to *Octβ2R*(+) cells, we find that optogenetic activation of *Octβ1R*(+) and *Oct-TyrR*(+) cells induce lateral oviduct contractions that are statistically different from the control. Stimulation of 2 of 5 preparations using the *Octβ3R* MiMIC driver was followed by lateral oviduct contractions (Fig. 7), but this did not statistically differ from the control. One presumably spontaneous contraction was seen during one of the stimulation periods for each of the controls that expressed *Octβ1R*-*MiMIC-Gal4*, *Oct3R*-*MiMIC-Gal4*, or *UAS-ChR2-XXM* alone (Fig. 7).

Although the oviduct epithelium expresses both *Octβ2R* and *OAMB*, we have previously shown that optogenetic stimulation of epithelial cells has no detectable effects on oviduct muscle contractions (Deshpande et al. 2022). In addition, we do not detect expression of any OA receptors in the muscles of the reproductive tract (Figs. 1–4 and Deshpande et al. (2022)). We therefore conclude that cells expressing *Octβ1R and Oct-TyrR* that respond to optogenetic stimulation are likely to be neurons, although it is not possible to rule out the possibility that receptor expression undetectable by the MiMIC lines but expressed at low levels in muscle could contribute to this response.

### Bath applied OA elicits calcium changes in sperm storage organs

**Fig. 8.**
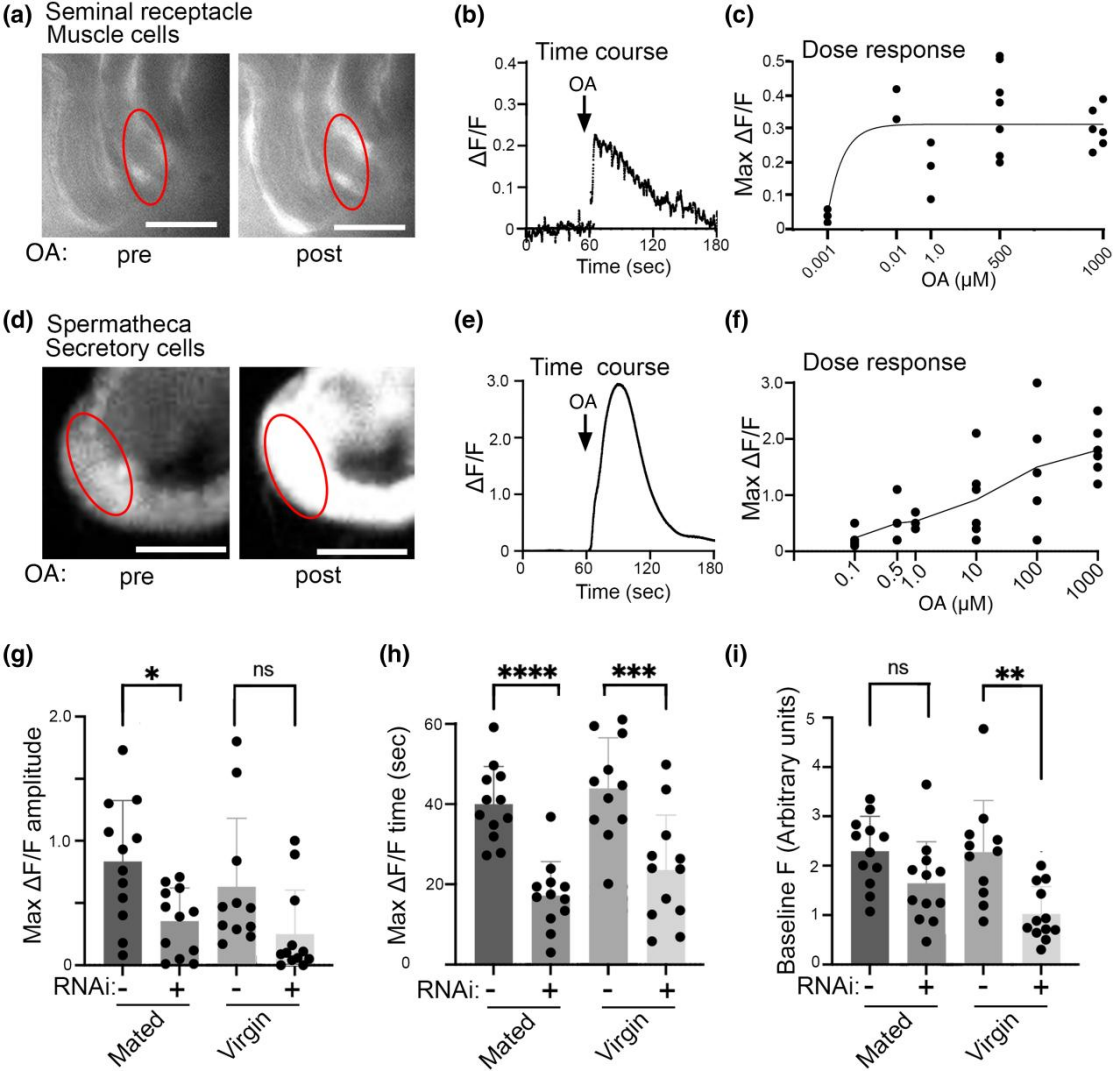
OA induces calcium transients in sperm storage organs. *UAS-RCaMP1b* was expressed in either muscle cells in the seminal receptable with *24B-Gal4* (a–c) or in spermathecal secretory cells with *40B09-Gal4* (d–i) followed by application of OA or vehicle. The red ovals represent examples of ROIs used for quantitation. Sample images (a) of seminal receptacle muscle cells pre- and postaddition (maximal response) of 1 μM OA at 60 s and a trace showing the time course of the response (b). A dose response plot shows seminal receptacle muscle cells increase intracellular calcium in response to OA doses ranging from 0.01 to 1000 μM (c). Sample images (d) of spermathecal secretory cells pre- and postaddition of 1 μM OA at 60 s and the time course of the response (e). Spermathecal secretory cells increase intracellular calcium in response to OA doses ranging from 0.5 to 1000 μM (f). Knocking down *OAMB* in the spermathecal secretory cells with *UAS-OAMB-RNAi* (indicated as “RNAi: +”) reduces the maximal RCaMP response to OA in the spermathecal secretory cells of mated flies (g), the time to maximal ΔF/F response in both virgin and mated flies (h), and the baseline RCaMP signal in virgin flies (i, arbitrary units × 10^3^) relative to matched controls (controls express *40B09-Gal4*, *UAS- RCaMP1b*, and *UAS-dicer2* but not *UAS-OAMB-RNAi*. Mean+/− SEM shown; 1-way ANOVA with multiple comparison test across all conditions within each panel, *P ≤ 0.05, **P ≤ 0.01, ***P ≤ 0.001, ****P ≤ 0.0001. Scale bars = 50 μm.

Bath applied OA induces muscle contractions and calcium transients within the muscle cells of both the ovaries and the lateral oviducts in *Drosophila* (Middleton et al. 2006) (Deshpande et al. 2022) (Meiselman et al. 2018). Muscle cells labeled with the *24B-Gal4* driver (Martinez-Azorin et al. 2013) also surround the lumen of the seminal receptacle. To determine if OA could stimulate the activity of muscles in the seminal receptacle, we used *24B-Gal4* to express the calcium sensor *UAS-RCaMP1b* (Fig. 8a–c). We observed frequent spontaneous calcium transients at baseline in the absence of OA (Supplementary Video 1) and an increase in calcium within seconds of applying of OA (Fig. 8a and b and Supplementary Video 1). The calcium transients appeared as waves, both at baseline and in the presence of OA (Supplementary Video 1). Dose response experiments indicate that the seminal receptacle muscle cells show a maximal response to OA concentrations as low as 0.01 μM (10 nM).

### OAMB regulates calcium transients in secretory cells within the spermatheca

The secretory cells in the spermathecae have been suggested to be regulated by OAMB (Avila et al. 2012) and previously shown to be involved in sperm storage (Schnakenberg et al. 2011) (Allen and Spradling 2008). As shown above (Fig. 6), we find that *OAMB* is expressed in the spermathecal bulb at a site consistent with previously described secretory cells (Schnakenberg et al. 2011) (Allen and Spradling 2008). To determine the acute effects of OA on the presumptive secretory cells of the spermathecae, we expressed *UAS-RCaMP1b* using the driver *40B09-Gal4* (Fig. 8d) and bath applied OA (or vehicle) to preparations of the reproductive tract that had been dissected out of the abdomen and both the CNS and all other surrounding tissue removed as described (Deshpande et al. 2022) (“isolated preparation,” see Materials and methods). We observed a robust increase in cytosolic calcium (Fig. 8d) that returned to a value near baseline within ∼80 s (Fig. 8e). Dose response experiments show that the secretory cells responded to OA at concentrations as low as 0.1–0.5 μM (100–500 nM; Fig. 8f), although the response was variable across preparations.

To directly assess the contribution of *OAMB* to calcium transients in spermathecal cells, we used a previously tested RNAi transgene directed against *OAMB* mRNA (Perkins et al. 2015). We tested both virgin and mated flies since a number of physiological changes occur after mating (White et al. 2021a) (Findlay et al. 2014) (Chapman et al. 2003). We again used the *40B09-Gal4* driver to express *UAS-RCaMP1b* and coexpressed *UAS-OAMB-RNAi* and *UAS-dicer2* to knock down *OAMB.* The controls for these experiments used *40B09-Gal4* to express *UAS-RCaMP1b* and *UAS-dicer2* in the absence of *UAS-OAMB-RNAi*. *OAMB* knock-down significantly blunted the maximum amplitude of the RCaMP signal seen in response to OA in the spermathecae of mated flies compared with controls; this effect was not statistically significant for virgin flies (Fig. 8e). Both mated and virgin flies showed a decrease in the duration of the calcium signal seen in response to OA (Fig. 8f, shown as the time from the onset of the increase until the maximum). In virgin, but not mated flies, the baseline RCaMP signal also appeared to be lower when *OAMB* was knocked down (Fig. 8i); however, we acknowledge the difficulty in assessing baseline fluorescence across different preparations. In sum, our data indicate that *OAMB* regulates the calcium response to OA of spermatheca secretory cells, consistent with the previously proposed role for *OAMB* in regulating sperm storage and suggesting that OAMB calcium-mediated pathways may be responsible for its effects on sperm storage (Avila et al. 2012) (Chen et al. 2022).

## Discussion

To gain insight into the mechanisms by which OA and cells that express OA receptors may regulate egg-laying, we have used a panel of high-fidelity Gal4 “MiMIC” lines to map expression in the reproductive tract of all the known *Drosophila* OA receptors. We also show that a subset of OA receptor–expressing cells may influence oviduct muscle contractions and that *OAMB* regulates the acute response to OA of a sperm storage organ.

We have previously shown that *OAMB* and *Octβ2R* are expressed in neuronal processes and peripheral neurons within the reproductive tract (Deshpande et al. 2022). Here, we show that multiple cells that localize to the reproductive tract also express *Octα2R*, *Octβ1R*, *Octβ3R*, and *Oct-TyrR*. Coexpression with the neuronal marker *ppk1.0-LexA* indicates that the cells that express *Octα2R*, *Octβ1R*, and *Octβ3R* in the reproductive tract are neurons. While we have not demonstrated colocalization of *Oct-TyrR(+)* with *ppk1.0*, the morphology of *Oct-TyrR(+)* cells and their processes within the reproductive tract strongly suggest that they, too, are neurons.

Potential differences between the functions of neurons that express each OA receptor subtype are highlighted by differences in the degree to which they appear to innervate each organ within the reproductive tract. For example, *Octβ1R*, *Octβ3R*, and *Oct-TyrR* innervate the base of the ovaries, suggesting the possibility that they could influence egg maturation. *OAMB*, *Octα2R*, and *Oct-TyrR(*+) processes innervate the SR, suggesting that both *Octα2R* and *Oct-TyrR* might play a role in sperm storage in addition to the established role for *OAMB* (Avila et al. 2012). All 6 OA receptors innervate the lateral and common oviducts, but the morphology of boutons at each site show subtle differences, and this too may influence their function. The morphological differences between glutamatergic, octopaminergic, and peptidergic boutons at the larval NMJ are well characterized (Jia et al. 1993) (Keshishian et al. 1996). The relationship between bouton morphology and neurochemical identity at other sites in the fly has received relatively little attention.

Nonneuronal cells that express *OAMB* in the reproductive tract include follicle cells that surround the developing oocyte and epithelial cells that line the lumen of the oviducts (Deady and Sun 2015) (Lee et al. 2003). We have confirmed that cells within the SR also express *OAMB* (Lee et al. 2003) and show that Ca^2+^ levels in the muscle of the SR are sensitive to OA. It possible that *OAMB(+)* cells within the lumen of the SR contribute to the response of the surrounding muscle, similar to the mechanism previously proposed for epithelial cells in the oviducts (Lee et al. 2009) (Lim et al. 2014). However, it is also possible that neuronal processes that express *OAMB* are responsible for this effect and further experiments will be needed to distinguish between these possibilities.

Previous results indicate that loss of *Octβ2R* blocks contraction of the lateral oviducts and optogenetic activation of *Octβ2R*-expressing neurons can induce lateral oviduct contractions (Deshpande et al. 2022). We find that optogenetic activation of *Octβ1R*- *and Oct-TyrR*-expressing cells can also induce lateral oviduct contractions. Since mutation of *Octβ2R* essentially blocks contractions caused by bath applied OA (Deshpande et al. 2022), the possibility that multiple, equally important, parallel pathways within the reproductive tract mediate oviduct contraction seems unlikely. Rather, we hypothesize that signaling pathways upstream of *Octβ2R* are responsible for these effects seen when activating *Octβ1R(+)* or *Oct-TyrR(+)* cells.

Previous studies have demonstrated a requirement for OA in the regulation of sperm storage by the spermathecae (Avila et al. 2012) (Chen et al. 2022), but the more acute effects of octopaminergic signaling at this site have been less clear. We show that OA induces calcium transients in secretory cells of the spermathecae and that this effect is blocked by knock-down of *OAMB* within these cells. These data suggest that calcium-mediated pathways may be responsible for the effects of OAMB on sperm storage. Since a secretory cell driver was used to knock-down *OAMB*, these effects would appear to occur via a cell autonomous mechanism within the secretory cells themselves rather than another cell type.

Most previous studies on reproduction in the fly have focused on the function of OA receptors in nonneuronal tissues, such as the epithelium and follicle cells (Li et al. 2015) (Lee et al. 2009) (Lim et al. 2014) (Deady and Sun 2015) (White et al. 2021b). Similarly, as noted above, we believe that nonneuronal cells regulate calcium transients in the secretory cells of the spermathecae and the oviduct epithelium as well as follicle cell rupture. Conversely, for the optogenetic stimulation of oviduct contraction, we suggest that effects we observe result from the activation of neurons or neuronal processes that express OA receptors. Both *OAMB* and *Octβ2R* are expressed in epithelial cells in the reproductive tract, and their expression in the epithelium is required for fertility (Lee et al. 2009) (Lim et al. 2014). However, expression of ChR2-XXM in epithelial cells does not induce oviduct contractions (Deshpande et al. 2022). In addition, we do not detect any of the 6 OA receptors in muscle cells using the GAL4 drivers (Deshpande et al. 2022) and we do not detect *Octα2R*, *Octβ1R*, *Octβ3R*, or *Oct-TyrR* in the epithelium, follicle cells, secretory cells, or any other identified nonneuronal cell types in the reproductive system using the MiMIC Gal4 lines. We therefore conclude that at least some of the effects we have observed when activating or inhibiting OA receptor–expressing cells are likely to be neuronal in origin. We anticipate that further experiments targeting subsets of OA expressing neurons will allow us to more determine the identity of these cells and thus increase our understanding of the complex neuronal network that regulates egg-laying.

## Supplementary Material

jkae012_Supplementary_Data

## Data Availability

Fly strains are available upon request and/or from the Bloomington Stock Center as indicated. The authors affirm that all data necessary for confirming the conclusions of the article are present within the article, figures, and tables. Full data sets are available on request. Supplemental material available at G3 online.
