# Supplementary material for: Expression and potential regulatory functions of Drosophila octopamine receptors in the female reproductive tract
Source: G3 (Bethesda). 2024 Jan 19;14(3):jkae012. doi: 10.1093/g3journal/jkae012 (PMC10917510; doi:10.1093/g3journal/jkae012)
Supplement: jkae012_Supplementary_Data [file jkae012_supplementary_data.zip › Supplementary_Video_1_Legend_G3-2023-404756.docx]

**Supplementary Video 1. Transient calcium wave activity in the muscle of the seminal receptacle.** RCaMP1b fluorescence in the muscle of the seminal receptacle presents as waves of increasing signal spontaneously both before and after the addition of Oa. A global increase in the amplitude of the RCaMP1b signal response is seen after addition of OA (1μM) at 5 sec. We did not detect a change in the rate of Ca^2+^ transients. The clip has been sped up 3x (12fps to 36fps) and trimmed to show only the 10 sec period bracketing the addition of OA at 5 sec.
